# An exploratory study of the socio-cultural risk influences for cigarette smoking among Southern Nigerian youth

**DOI:** 10.1186/1471-2458-14-1204

**Published:** 2014-11-22

**Authors:** Catherine O Egbe, Inge Petersen, Anna Meyer-Weitz, Kwaku Oppong Asante

**Affiliations:** Discipline of Psychology, School of Applied Human Sciences, College of Humanities, University of KwaZulu-Natal, King George V Avenue, Durban, 4041 South Africa

**Keywords:** Youth, Cigarette smoking, Cultural practices, Tobacco policy, Nigeria

## Abstract

**Background:**

The increase in smoking prevalence in developing countries including Nigeria has been mainly blamed on the aggressive marketing strategies of big tobacco companies. There is a paucity of research on other socio-cultural risk factors for smoking among the youth. The main objective of this study is to explore and describe socio-cultural risk factors influencing cigarette smoking among the youth in Southern Nigeria.

**Methods:**

A total of 27 respondents (5 community leaders, 4 political analysts and 18 young cigarette smokers) were interviewed using a semi-structured interview guide. Interpretative Phenomenological Analysis (IPA) was used to analyse the data.

**Results:**

Social-cultural practices fuelling early usage and exposure of children to cigarettes and the promotional activities of tobacco companies were identified as possible factors influencing youth’s smoking behaviour in Southern Nigeria.

**Conclusion:**

Tobacco control policies should include cultural interventions to modify current traditional practices and social norms which fuel the use of tobacco in the society. Such interventions must target specific groups, subpopulations and subcultures more exposed to the cultural risk influences for smoking.

## Background

The World Health Organization (WHO) estimates that nearly six million people die of tobacco related diseases around the world annually [1]. Despite efforts aimed at reducing the number of people picking up the habit of smoking, prevalence rates are still high in many parts of the developing world especially in Africa and Asia [1, 2]. Sub-Saharan Africa accounts for 2% of the world 11% population who are smokers [3].

There are conflicting evidences on the actual prevalence of cigarette smoking in Nigeria. Individually conducted studies in parts of Nigeria show a considerably high smoking prevalence rate for example; 17.6% obtained among rural dwellers in the South-west region in 2003 [4] and 31.9% among adults in North-Eastern Nigeria in 2008 [5]. The prevalence rate obtained from the national survey carried out in 2002 was 8.6 [6]. Results from the Nigeria demographic and health survey (NDHS) 2008 found that 3.06% of adults aged 18 to 29 years and 9% of adults aged 15–49 years smoke cigarette [7, 8]. The World Report on the Global Tobacco Epidemic [9] shows smoking prevalence among Nigerian youth to be 3.5% as at 2008. A more recent Global adult tobacco survey (GATS) carried out in 2012 found that 3.7% of adults aged 15 years and above smoke cigarette in Nigeria [10]. In the face of these conflicting reports however, Drope reports that the smoking prevalence among adolescents and young adults in Nigeria is on the increase [11] just like in many developing countries as mentioned earlier.

The increase in smoking prevalence in developing countries (including Nigeria) has been mainly blamed on the aggressive marketing strategies of big tobacco companies [3, 12]. However, many aspects of tobacco use are controlled by culture [13]. The tobacco plant and tobacco products like snuff and cigarettes are also symbolic in many traditions across the world [14, 15]. Meanings surrounding tobacco use differ across cultures. Early societies have viewed tobacco smoking as a symbol of hospitality, communication with the gods and spirits and possessing the healing power to chase away diseases [15]. Some cultures view tobacco smoking as a prestigious behaviour resulting in great personal satisfaction [16]. Studies have also shown some cultural explanations of smoking behaviours across some ethnic groups within and outside Nigeria [13, 14, 17]. In a study of five ethnic groups across eleven states in the USA, Mermelstein and colleagues [17] found striking differences across ethnic and gender sub groups in reasons for not smoking. Further studies in Asia also found culturally specific contexts for smoking among Bangladeshi and Pakistani adults with tradition, culture and the family playing significant roles in nurturing and cultivating norms and values around smoking [18]. In a study carried out in North-Eastern Nigeria, it was found that being of the Margi, Hausa and Fulani Ethnic tribes were strongly associated with smoking [6].

These aforementioned studies indicated the need for further investigations of this seemingly cultural phenomenon [19, 20]. However, there is a paucity of research in this area within Southern Nigeria. We are therefore not fairly informed about the socio-cultural risk factors for smoking among the youth in Nigeria. The main objective of this study is to explore and describe the socio-cultural risk factors influencing smoking among young smokers. The specific question addressed in this study is: what socio-cultural factors influence smoking among the youth in Southern Nigeria? Findings from the present study provide a phenomenological overview of risk factors for smoking in Nigeria. Findings will further facilitate policy development of tobacco control interventions sensitive to the contexts within which smoking is initiated and perpetuated among the youth.

## Methods

### Design

The qualitative design was considered appropriate for this study as it allows the researchers to capture the words, perceptions and experiences of participants and for “thick and rich” descriptions of the issues under study [21].

### Participants and setting

This study was carried out in southern Nigeria, which has seventeen states with only one (Osun state) having a functional state-enacted tobacco control policy [11]. Southern Nigeria is divided into three geopolitical zones (south-east, south-south and south-west zones). South-eastern Nigeria is home to the Igbo ethnic nationality. South-western Nigeria is home to the Yoruba ethnic nationality while the South-south has a diversity of smaller ethnic nationalities including the Ijaw, Urhobo, Edo, Itsekiri and Ibibio ethnic groups among others. Participants were a purposive sample of 27 community members (using snowball sampling technique) in three categories namely; 5 community leaders (sampled from the three geopolitical zones in southern Nigeria), 4 political analysts/NGOs officials (PA) and 18 young male smokers (YS) aged between 18 and 24 years (mean age of 23 years). This sampling technique was appropriate as it enables the researchers to recruit selected participants according to their ability to provide rich information on the phenomenon under study [22]. All the young tobacco users were males. They include; Young smoker (YS) university undergraduates (YS-US: n = 3); ‘other students’ i.e. students of other tertiary institutions aside the University (e.g. Colleges of Education, Polytechnics etc.) (YS-OS: n = 4); skilled workers (YS-SW: n = 5) and unskilled workers (YS-USW: n = 6). The mean age of smoking initiation was 15.2 years. Majority of young smokers (n = 12) have had education up to the secondary school level, and all participants had a close friend who smokes. Seventeen out of the 18 young smokers had a family relative who smokes and the same number of young smokers had attempted to quit smoking. Community leaders are regarded as the custodians of culture in traditional Nigerian settings. They are sometimes appointed to lead or could be the oldest man in the community as is most often the case in South-south Nigeria. Community leaders were purposively sampled from Anambra, Edo and Ondo states in Southern Nigeria.

The inclusion of the three categories of respondents served the purpose of triangulating the data obtained in this study and the reduction of bias in interpretation of participants’ responses. This was ensured by including in the three interview schedules used to guide the interview, some questions cutting across the different groups of respondents.

### Data collection

Data were collected by the first author by means of twenty-four individual interviews and one small focus group discussion comprising of three community leaders (from south-west Nigeria). The interview with the South-west community leader turned out to be a focus group discussion involving three participants because two other elders of the community who were visiting the community leader (earlier contacted) at that time also volunteered to participate in the interview since the discussion concerned their cultural practices. All interviews with community leaders took place in their respective communities (in their residences) while the interviews with young smokers took place at various locations in which each of the participants were more comfortable with. Worthy of note is the fact that more than half of the young smokers interviews preferred locations which protected their identity. The interviews were guided by semi-structured interview schedules. In addition, young smokers also completed a semi-structured biographic questionnaire assessing their bio data and smoking history. The interview with the young smokers asked about the role of specific cultural practices and personal and social factors in their tobacco use. Political analysts answered questions bothering on socio-political trends affecting the issue of tobacco policies. Community leaders were required to provide answers to questions mainly concerning the cultural symbolism of tobacco and tobacco use in their community. Interviews were mostly conducted in the English language and Nigerian Pidgin English spoken as an informal *lingua franca* across most parts of Nigeria. Three other indigenous languages (Igbo, Yoruba and Edo) were also used in some parts of the interviews with the community leaders. Interpreters were used where the language spoken was not English or the Nigerian Pidgin English. The interview schedules had questions such as: How did you start smoking? (For young smokers only); are you aware of promotional activities of tobacco companies in Nigeria? Are there cultural practices/ceremonies in your community which involve the use of tobacco and/or tobacco products e.g. snuff, cured tobacco leaves, cigarettes, etc.? If yes, please mention them; Please describe the role tobacco or tobacco products serve in these ceremonies (if any) and explain how it is consumed; Do the youth participate in any of these ceremonies? How? What part of your culture do you think influences the youth to smoke? How do you think tobacco companies influence the youth to smoke in Nigeria? What do you think the government of Nigeria has done concerning the issue of smoking among young people?

### Ethical consideration

The University of KwaZulu-Natal Research and Ethics Committee granted ethical approval for this study (Ethical Approval number: HSS/1485/010D). Through an informed consent form, participants were enlightened about the aims of the study and of their freedom to participate or withdraw from the study at any time. They were also assured of confidentiality and anonymity of their identity in the publication of their responses. Participants signed the informed consent form as a proof of this agreement.

### Data analysis

Transcription of the recorded interviews was done in the language the interviews were conducted after which those needing translation were translated into English language. Data were thematically analysed using the four steps of IPA [23]. Coding of the data was informed by *a priori* concepts [23] in the interview schedule as well as new emergent themes from the interviews. In the first step, the authors read and re-read the transcribed data to familiarise with the data and made notes. The second involved harmonizing, identifying and labelling themes from the notes that had been identified by the authors. Connecting the identified themes and merging of appropriate sub-themes to form a main theme was done in the third stage. In the final analysis, we summarised the main themes with their sub-themes, with supporting illustrative quotations before writing it out in a narrative form. Two independent coders were engaged to cross-validate the emergent themes. The software Nvivo 9 was used in the data analysis.

## Results

Using Interpretative Phenomenological Analysis (IPA), two broad themes were identified. These are: *The cultural environment* which describes the nature of cultural practices, trends and norms that influence youth smoking behaviour. The subthemes identified include; primal culture involving the use of tobacco products, socialization practices, social and perceived norms for and against smoking, emerging cultural practices. The second theme is the *Policy environment.* Here the effects of a non-comprehensive and largely non-operational tobacco law are explored and elucidated. Three main sub-themes that emerged were; non-operational tobacco laws, availability of cigarettes and the influence of the activities of tobacco companies.

### Cultural environment

#### Primal culture involving the use of tobacco products

Cultural practices that require the provision of cigarettes and tobacco related items for traditional ceremonies such ]as burials and marriages are prevalent in Southern Nigeria. Findings in this study revealed that of the three regions in Southern Nigeria, tobacco use seems to have the strongest cultural symbolism in south-eastern Nigeria. A south-eastern community leader explains how tobacco is used in his culture in the narrative below:

*There are many ways we use it as part of our culture… especially in the area of marriage. If one is giving a daughter in marriage, there are things the suitor must do. This cigarette, tobacco, that is, the cured leaves and the other ingredient…they call ‘akanwu’ and other things…you must present them for the marriage to your in-laws. Should the person refuse to provide the cigarette, the youth will spoil that marriage ceremony. The cigarette must be there! (South-east community leader, Male)*

The narrative above underlies the cultural importance of tobacco in this community. The absence of such items in marriage ceremonies could be considered as unacceptable and possibly lead to the postponement or disruption of the marriage ceremony. While the cultural use of tobacco seems to be reducing in south-south Nigeria, the use of snuff by old men in the south-west and the use of cigarettes and other tobacco products in many parts of south-eastern Nigeria were found to still persist.

#### Socialization practices

Respect for older members of the community was found to serve as a risk influence for smoking in a collectivist culture such as that which exists in Nigeria. It is common practice for older members of the community to send children and older adolescents on errands to purchase cigarettes. Sometimes, they are asked to light up the cigarette at the point of purchase. Participants explained that once a child says he was sent to buy cigarettes by an older adult, he is also not questioned at the sales point since this is a common practice. The following quotes are illustrative of this:

*In my community…there is this…would I say, like a culture there that you must respect your elders. The ones that are senior to you in the town… if they ask you to go and do anything for them, you will go ahead and do it for them or else they will think you are disrespecting them. I remember there was this kid that I saw in my compound who was not up to 9 years old…. Someone had sent him to go and buy cigarette. Behold, I saw this little boy trying to smoke the cigarette. I then shouted “stop it!” The boy was shocked. Then I asked “who sent you?” He said “bros” [a name called older males]….“now take it to bros” I said. Then I said to myself; you know by the time they keep sending this boy there is going to be a time when he would like to…he would like to say, “okay madam, give me Benson” [telling the seller]. When he is asked “Who sent you?” he would say “bros”. But unknowingly to the seller, the cigarette belongs to the kid. He will look for anywhere just to hide and to smoke it the way ‘bros’ does…you know…(YS-Skilled Worker 1, Male; 24 yrs)*

This narrative indicates that the cultural norm of obedience and unquestioned respect for older members of the community including the buying of cigarettes has a negative influence on children. By this practice, children and older adolescents are thus exposed to a culture of cigarette smoking from an early age. This practice also makes cigarettes accessible to children and adolescents since they can easily disguise to buy cigarettes for their own consumption.

#### Social and perceived norms for and against smoking

The society is seen to send contrasting messages on the use of cigarettes and other tobacco products among various age groups and genders. For example, while it is regarded as a thing of pride for older men and women to use tobacco in the form of snuff or to smoke its dried leaves in pipes (more prevalent in the past), tobacco use in the form of cigarette smoking by the youth has always been frowned upon by older adults. It is perceived as an irresponsible behaviour and a sign of deviancy. For females, it carries an even more negative connotation. It is therefore understandable why many young smokers conceal their smoking status.

*Not that they [smokers] are not really good people. They [the society] see it as though you are not responsible. They don’t see it [smoking] as a good sign. (YS-Skilled Worker 3, Male; 23 yrs)*

*Yes…a lot of adults are smoking. You know like in Africa and Nigeria particularly what the adults do that may be wrong and injurious to their health, the youth or children are not allowed to do the same. (Political Analyst 2, Female)*

#### Emerging cultural practices

Emerging cultural practices refer to current practices that are fast becoming as strong as primal cultures. These are greatly influenced by media exposure or exposure to other cultures. The provision of cigarettes at some cultural ceremonies like burials is an example of an emerging cultural practice. This tradition was not initially part of the indigenous culture of these people but is being recently introduced in such communities and has become part of the norm at these ceremonies in some parts of southern Nigeria as explained in the following narrative.

*There was one ceremony which they had the other day…even though it is compulsory according to their…I don’t know, it was not the real community traditions that actually introduced that law. That law or that tradition was introduced by the age group. Any time they want to bury someone…they demand some items like two packets of cigarettes, two cartons of drinks, kola and some other things. Like the other day we buried one of our aunties they demanded two packets of cigarettes…they insisted that I must provide that cigarette (Political Analyst 3, Male)*

The media through adverts and movies have also promoted norms among the youth that suggest smoking to be a “cool” thing to do. Media depictions portray smoking as an identity for the successful or the “upward mobile”; a sign of adulthood, independence and toughness. This media depiction of smoking has become more acceptable to the youth than any form of health promotion message concerning tobacco use. This is expressed by the following narrative:

*The impression they [the media] create is that…they make you believe that when you smoke, you are a big boy. You are a hard guy…a kind of person that makes things work…make things tick…something like that (YS-Undergraduate Student 2, Male; 23 years).*

One smoker described how he would stop a movie to go have a cigarette on seeing smoking scenes;

*“…I would say “this is the Don. I love the way the guy holds the cigarette and the way the guy does his things” and after watching the movie…wow!…most of the time I even put off the movie, stop it there just to go and have a stick of cigarette (YS-Skilled Worker 1, Male; 24 years).*

### Policy environment

#### Non-comprehensive and non-operational tobacco laws

In Nigeria, there has been one tobacco law promulgated in 1990 by the then Military government: the Tobacco Smoking (Control) Decree 20. This was later converted to an Act during democratic rule and it was titled “Tobacco (Control) Act 1990 CAP.T16” [11]. This law as noted by Drope [11] and participants in this study, is presently not being implemented in its entirety and in the entire country.

*I don’t know about its enforcement but the law is there, it is existing. At the beginning, they were trying to enforce it.…it is not being implemented because if you went to public places then…they followed it up… you couldn’t smoke in public transport, may be you are in a taxi, you couldn’t smoke. In a school like this, you couldn’t smoke but now people smoke because there is really no follow-up on implementation (Political Analyst 2, Female).*

Reasons for the non-implementation of the law include lack of awareness of the law as a result of it not being enforced. This in turn is a product of a perceived lack of importance of the law on the part of government and pretense of ignorance on the part of law makers which could be as a result of a lack of commitment to ensure the well-being of the citizenry. Narratives supporting this assertion are indicated below:

*Maybe because there was not enough awareness…not enough people knew…It was weak and then there was nobody that was really pushing it…It wasn’t enforced, it was not really effective (Political Analyst 4, Female).*

*Well…non-implementation could be from different angles… Because there is no social structure, no system in place, people get away with not obeying laws…nobody actually feels threatened so nobody feels obliged to implementing them…there is no agency saddled with the responsibility. And if it is not carried out, there is nobody you can hold…and I don’t think the sanctions too were enough though… I can’t even remember what the sanction was so that’s to tell you how weak the law was…it was very weak…So these people in positions of authority, either they don’t have a good grasp of the importance or the effect of not passing this new law or this new bill into law or they pretend not to know (Political Analyst 2, Female).*

Among young smokers there was a general low level of awareness about the past law and current efforts at promulgating tobacco control laws in Nigeria. The youth were however aware of the age restriction and health warnings imprinted on cigarette packs but noted that the age restriction for tobacco sales is not being enforced. This therefore makes it very easy for children and youth to access cigarettes.

*The only thing I’m aware of is that they will just say smokers are liable to die young. That is all they do. (YS-Undergraduate Student 3, Male; 24 yrs)*

*Yeah…if there is probably a law for that, we don’t implement that because you see a child of 2, 3 yrs he or she will be sent to go and buy cigarette. (YS-Skilled Worker 2, Male)*

### Availability of cigarettes

Generally, the availability of cigarette is largely controlled by pricing and tobacco control laws. Availability can also refer to proximity of points of sale and ease of purchasing cigarette within the community. Cigarettes are widely accessible in Nigeria. They are sold in stores and kiosks and houses, making it easy to purchase it at any time of the day in residential areas. This is especially worsened due to the non-enforcement of age restrictions in purchasing cigarettes. One participant explains that many sellers of groceries sell cigarettes to promote their business since it is a fast selling item.

*Around my area…I told you it’s a common phenomenon around here. Nearly all the kiosks, all the shops around here sell cigarettes. (YS-Undergraduate Student 2, Male; 23 yrs)*

*Just like the age of my boy [referring to his three year old son playing in the courtyard]…I can give him an empty cigarette packet and ask him to give the woman who sells at the counter [a kiosk] so that she could sell cigarette to him. (South-south Community Leader, Male)*

Cigarettes are also sold during social ceremonies like burials, marriages and cultural festivals. Participants indicated that this was another pathway for youth to access cigarettes. Cigarettes are being sold in single sticks and this serves the same purpose of making it easily accessible to young people as well.

*Yeah …there is no occasion you will go to, that you will not see hawkers or people with tables selling cigarettes on them. (YS-Skilled Worker 3, Male; 23 yrs)*

### Influence of the activities of tobacco companies

Findings suggest that tobacco companies are capitalizing on the defunct nature of the Nigeria’s Tobacco Control Act of 1990 and a yet to be passed Bill on tobacco control to carry out promotional activities which they have been banned from doing in many other countries.

In diverse ways, the presence of tobacco companies and their efforts at staying in business have influenced the relatively high prevalence of smoking amongst the youth. More than half of the young smokers interviewed mentioned that they still saw tobacco adverts in different forms of media, especially on bill boards and the print media. Television adverts, however, were said to have been stopped a few years back. Moreover, some participants remember quite vividly the words of such adverts and the effect they had or still have on them.

*Because when I was growing up, there were still these things then…there was still this St. Morris advert on TV…it was so, so spectacular… The guy was too cool, well dressed…in a very good house, good car and everything and at the end of the day he topped the whole thing with a stick of cigarette. So it was as if the VIPs smoke. (YS-Undergraduate Student 2, Male; 23 yrs).*

Other participants indicated that, subtle, yet strong adverts which have proven to be effective ways of getting more youth to smoke are increasing in Nigeria. They are aggressively done through services such as scholarships, organizing promotional events and parties for the youth and fashion shows where cigarettes are made freely available. Some narratives of this observation are presented as follows:

*In the university, I had friends that went to write a scholarship exam that was being given out by these…tobacco companies. And where did they write this exam? In the tobacco company’s premises! And there were cigarettes lying around everywhere (YS-Undergraduate Student 2, Male; 23 yrs)*

*They had these fashion shows…it was one of their strategies to encourage women to smoke; the St Morris Fashion Show. Incidentally, I was a reporter in News watch [one of Nigeria’s National weekly magazines] and I covered about three of the events…you go to the fashion show and they put cigarettes on all the tables and everybody will be smoking (Political Analyst 4, Female).*

A summary of the socio-cultural risk influences for smoking found in this study is presented in Table 1.

**Table 1:** **A summary of socio-cultural risk influences for youth smoking behavior in southern Nigeria**

Cultural environment	Policy environment
Primal culture involving the use of tobacco products	Non-comprehensive/non-operational tobacco laws
Socialization practices	Availability of cigarettes
Social and perceived norms for and against smoking	Influence of the activities of tobacco companies
Emerging cultural practices	

## Discussion

The main objective of this study was to explore socio-cultural risk influence for smoking among the youth in Southern Nigeria. The findings have shown that cultural factors (such as primal culture involving the use of cigarette, socialization practices, social and perceived norms for and against smoking and emerging cultural practices) and the policy environment (e.g. non-comprehensive/non-operational tobacco laws, activities of tobacco companies and availability of cigarettes) within the study area were the possible risk factors for cigarette smoking among the youth.

Culture was found to play a crucial role in increasing youths’ access to cigarettes, as a result of existing indigenous cultural practices in parts of southern Nigeria which demand the provision of cigarettes at cultural ceremonies like marriages and burials. This supports the studies of Feinhandler [15] and Ding and Melbourne [24] which have reported the use of tobacco products for cultural purposes. Community leaders, political analysts and NGO officials indicated that with more political will on the part of government and elders, cultural practices involving the consumption of cigarettes and snuff can be replaced with less harmful items like the kolanut (*Cola acuminata* or *Cola nitida*) or alligator pepper (*Aframomum melegueta).* These other alternative items are culturally valued agricultural items in Nigeria and many tropical African countries [25]. It is however, important to note that the re-negotiation of cultures involving tobacco use in this context may not likely be achieved by just policy interventions, as traditions are engrained within indigenous cultures in the community [26]. Decisions involving changes in these traditions and practices are not usually done at an individual or group level, but rather through a negotiated process involving the traditional political structures of traditional kings, chiefs and council of elders [26]. This pathway to review traditional practices can also be used to discourage parents who smoke and other community members from sending children on errands to buy cigarettes.

The findings of the study also showed that socialization practices can and do create an acceptance of tobacco from a young age due to the norm of sending minors to buy and/or sell tobacco products [27]. While it is culturally acceptable for children to do errands for older adults, sending them to buy cigarettes becomes a socialization process for them to normalize the use of tobacco products and actually experiment it. Over ninety percent of the young smokers (n = 16) interviewed had as minors been sent on such errands to buy cigarettes for older members of their community. However, as noted in Egbe et al. [27], this cultural trend will be difficult to change but this can be given a try by using traditional political structures.

Due to the ineffective nature of the existing but non-comprehensive tobacco control law and a new law still being processed (in the national legislative houses at the time of writing this paper), Nigerian youth come face to face with direct media messages which advertise smoking in a positive light and do not provide messages of the negative health consequences of smoking. While tobacco advertising was completely banned in the Nigerian media in 2002 by the Advertising Practitioners Promotion Control of Nigeria (APCON) [11], this seems to also lack enforcement as more than half of the participants reported that tobacco adverts still abound especially in the print media and bill boards. In addition, smoking scenes in local and international movies generously abound in Nigeria. The findings revealed that the characteristics of popular culture such as the creation of idols, stars or superheroes [28] through tobacco advertising were found to serve as risk influences for smoking in this study. A strong, direct and independent association has been found to exist between seeing tobacco use in films and trying cigarettes among a sample of adolescents [29]. This therefore suggests that individuals with higher exposure were significantly more likely to have experimented smoking. This can be supported by the narratives of a young smoker in this study who described how he would pause to have a stick of cigarette just by watching a movie actor smoke in the movie. This is also reflected in a South African study by Brook, Pahl and Morojele [30] which found that adolescents’ receptivity to media models of smoking is related to nicotine dependence.

Our findings also identified the easy access and low price of tobacco, the seemingly lack of government’s regulation of the activities of tobacco companies and emulation of adult role models who smoke as risk influences for cigarette smoking. Restricting the sale, purchase and increasing the prices of cigarettes have been found internationally to curb the smoking rate in South Africa, UK and the USA where these restrictions have been introduced [31–33]. Cigarettes are readily available and within reach to all individuals irrespective of age and location in Nigeria. This availability increases access to cigarettes by youth, encouraging them to initiate and perpetuate smoking.

The award of scholarships by tobacco companies to indigent students, especially those from tobacco farming areas, was also identified as an indirect tobacco promotional activity targeting the youth. A participant mentioned that scholarship exams are usually held in the work site of tobacco companies where cigarettes are made freely available. The representatives of big tobacco companies were said to have privately admitted that the purpose of their activities aimed at Cooperate Social Responsibility (CSR) is to protect their reputation and boost shareholders’ value [34]. Corporate Social Responsibility involves the provision of some basic physical and social amenities in communities, aids to farmers etc. carried out by the tobacco companies as a way of giving back to their host communities/governments in order to maintain their relevance in that environment. In this sense, CSR is used more as a marketing vehicle to boost consumption than for community development especially in Africa [35].

Tobacco advertising and promotion are aggressively carried out by tobacco companies in Nigeria. Three young smokers interviewed in this study reported attending tobacco promotional activities recently. Advertising and promotion are very effective tools in influencing young people to initiate and later become established smokers [36]. A significant association was found by Mowery, Farrelly, Haviland, Gable and Wells [37] between being receptive to tobacco industry promotions and being open to smoking. While Leatherdale, Sparks and Kirsh [38] also found that occasional and regular smoking behaviour is significantly linked to students’ belief about tobacco companies doing good things in the community. This they say manipulates young people to think that it is cool to smoke as was found in this study.

### Implications for tobacco control interventions

The findings in this study are relevant for tobacco control interventions especially within Nigeria. Pro-tobacco cultures (both primal and emerging ones) and weak policy environment currently impact on the increasing prevalence of smoking among the youth. In many countries, policy interventions have been successful in reducing smoking prevalence [32, 33]. However, in this context, it may be necessary that both cultural and policy interventions be synchronized to effectively achieve the desired outcome of reducing smoking prevalence especially among young people.

The establishment of an agency to co-ordinate tobacco control activities at all levels of government in Nigeria is paramount. It is important that health promotion practices as well as proposed policy interventions take into account the social identities of individuals when seeking to modify health risking behaviours which are engrained in the cultural environment especially in Africa due to the dominance of collectivist culture.

Theory-informed health promotion campaigns targeting the re-negotiation of social norms and cultural practices around the use of tobacco and tobacco products within the cultural environment are needed. In the African context, where traditions are preserved, controlled and transmitted through the traditional political structures, the role of traditional leaders in developing tobacco interventions cannot be underestimated [26]. Although, they are not traditionally empowered to effect a change in traditional cultures in their personal capacities, they are in a better position to initiate the process of change within the community. Primal cultures involving the provision and consumption of cigarettes and other tobacco products especially in South-eastern Nigeria should be re-negotiated to stop their use. Also, emerging cultures in South-south Nigeria which demand the provision of cigarettes and other tobacco products need to be monitored and nipped in the bud by the traditional political structures in such communities. There may be a need to established community-based tobacco control bodies who will monitor the implementation of such traditional laws if put in place.

Health education on the dangers of smoking should be designed to target specific groups, subpopulations and subcultures more exposed to cultural risk influences. This is important to make it easier for such populations to accept a change in culture that would be lifesaving for them in the near future.

### Limitations of the study

The small sample size and the non-probability sampling method (which is characteristic of qualitative studies) mean that the findings cannot be generalized to all young smokers in Southern Nigeria. While our sample of cigarette smokers is unintentionally biased towards male participants, this reflects the male dominance of smoking among the youth in Nigeria [11, 12]. Nevertheless, further studies should explore risk influences for smoking among female smokers which is a largely inaccessible population in Nigeria as was found during the data gathering process of this study [39]. Despite these shortcomings, which are generally associated with qualitative studies, the results presented fill a substantial gap in research that explore socio-cultural risk factors that influence smoking among the youth in the Southern part of Nigeria. Further studies can extend this study by focusing solely on how cultural factor influence tobacco use in Nigeria, and other similar cultures. In doing so, there would be the need to create a more comprehensive and cultural sensitive questionnaire which would collect adequate information on various aspects of cultural practices where tobacco or its products are used. Some risk influence for smoking such as unemployment and personality attributes of smokers which was not explored in this study could be good research areas for future studies.

## Conclusion

This study has shown that primal culture involving the use of tobacco products, socialization practices and emerging cultural practices, non-comprehensive/non-operational tobacco laws, activities of tobacco companies and availability of cigarettes were socio-cultural risk influences for cigarette smoking among the youth in Southern Nigeria. Tobacco control policies and efforts should include cultural interventions to modify current traditional practices and social norms which fuel the use of tobacco in the society.
